# Qualitative studies: designing a multimodal medical visualization tool for helping patients interpret 3D medical images

**DOI:** 10.3389/fphys.2025.1559801

**Published:** 2025-05-26

**Authors:** Jinjin Chen, Ji Ma, Yongjian Huai

**Affiliations:** ^1^ School of Design and Art, Communication University of Zhejiang, Hangzhou, China; ^2^ School of Ocean Information Engineering, Jimei University, Xiamen, China; ^3^ School of Information, Beijing Forestry University, Beijing, China

**Keywords:** interpreting 3D medical images, multimodal, medical visualization, qualitative research, design consideration

## Abstract

**Background:**

Accurate understanding of 3D medical images requires a background of specialized medical knowledge. There is a pressing need for easy-to-understand medical visualization tools to help patients accurately interpret 3D image data, especially given the large number of patients requiring such assistance.

**Objective:**

In this paper, we explore the design considerations of a multimodal medical visualization tool for interpreting 3D medical images, which can help users to understand and recognize 3D medical image data.

**Methods:**

An observational study and focus group interviews were conducted to explore how patients interact with physicians and the main problems they encounter when interpreting 3D medical images. Additionally, we conducted semi-structured expert interviews with physicians to investigate the common methods, techniques, and challenges involved in doctor-patient communication when interpreting 3D medical images. We also organized a participatory design workshop to discuss the patients’ design preferences for medical visualization tools.

**Results:**

The study identified three types of physician-patient interactions, eight specific behaviors, and seven main issues. It also summarized eight common methods and techniques to aid in understanding 3D medical images and highlighted five key findings regarding design preferences for medical visualization tools. Based on previous studies and our empirical research results, we propose seven design considerations for designing visual interfaces, interaction design plans, audios, infographics, and animation guides. The comprehensive summary of the weights for the above-mentioned design consideration was obtained. A comprehensive weighting of design consideration elements was calculated based on the Analytic Hierarchy Process. The results show that the design consideration factors (A primary factors) that have the relatively big weights are the interaction design (57.091%) and visual interface (25.352%), and the ones that have relatively small weights are the medical education and popularization (12.766%), and text presentation (4.791%). Additionally, we found that the weights of factors of the design considerations (B primary factors) are different in the web application, software and VR/AR platforms. Furthermore, we presented a case study of the design of a multimodal medical visualization tool applied in the medical context to help patients interpret 3D medical image data and improve doctor-patient communication skills.

**Conclusion:**

This study explores the benefits of combining multiple visualization methods for both doctors and patients. We also discuss the advantages and challenges of designing and using multimodal visualization tools in medical settings.

## 1 Introduction

Interpretation and understanding of 3D medical images are becoming increasingly important in medical education and practice, not only to the education and training of medical students (Beermann et al., 2010; Keedy et al., 2011; Estevez et al., 2010), but also for enhancing understanding and awareness for patient education (Morris and Van Wijhe, 2010; Phelps et al., 2017; Williams and Cameron, 2009). Accurate interpretation of 3D medical images significantly enhances diagnostic accuracy (Gao et al., 2022), improves patient compliance (Jones, 2018), and satisfaction (Vilallonga et al., 2012). When interpreting medical images, physicians in clinical practice commonly use two-dimensional images with medical indications, even though these images are derived from key slices of three-dimensional medical scans. Compared to 2D medical images, 3D medical images optimize information better (Fischer et al., 2008; Maleike et al., 2009) and are easier to understand spatial anatomy (Garg et al., 2001). Unlike physicians who are professionally trained and specialized in interpreting complex medical images, patients often have difficulty in understanding these medical images (Kessels, 2003; Ley, 1979). Several previous studies have indicated that patients prefer to simultaneously view 3D medical images during diagnosis (Phelps et al., 2017; Treisman and Gelade, 1980) because it not only can help patients comprehend and recall (Phelps et al., 2017), but also can enhance their understanding of disease and treatment (Morris and Van Wijhe, 2010; Devcich et al., 2014; Carlin et al., 2014). Studies have shown that the use of visual means (Williams and Cameron, 2009) such as pictures, diagrams, and 3D images can effectively help patients understand the medical knowledge (Morris and Van Wijhe, 2010; Williams and Cameron, 2009). Phelps et al.'s study found that 3D images, due to their easily recognizable structures, can significantly benefit a large number of patients by aiding their understanding (Phelps et al., 2017). When interpreting 3D medical images, a variety of medical visualization tools and interactive software play a crucial role. These tools include technologies such as VR, AR, mobile wearable devices, software, applications, electronic health records, and digital platforms, which are used to display medical information (Lupton et al., 2022). Some studies have preliminarily shown that medical visualization improves perception, understanding, and subsequent health behaviors (Jones, 2018). With the continuous advancement in medicine, 3D multimodal medical images have clearer and more precise image resolution (Polvara et al., 2019; Liu et al., 2021), which enable to effectively segment abnormal and normal tissues, and make doctors and patients to understand the disease more intuitively (Gao et al., 2022). Therefore, tools designed to interpret 3D medical images also need to be constantly innovated to eliminate the gap between doctors and patients when interpreting medical information. There are few studies on how to bridge the gap in patients’ interpretation of 3D medical images, increase the informed management of their own health status and their participation in the diagnosis and treatment process, as well as demonstrating the benefits of interpreting 3D medical images by patients who do not take professional training (Phelps et al., 2017).

This paper presents a methodology of interpreting 3D medical images to patients so as to facilitate effective communication and collaboration between doctors and patients. Additionally, it explores the design considerations of the 3D medical image tools which can help patients to interpret the images. The core research question (RQ) is what are the design considerations of a multimodal medical visualization tool for interpreting 3D medical images? Based on previous studies and our empirical research results, we propose a series of considerations for designing multimodal medical visualization tools and guide the design cases of multimodal medical visualization tools so as to help patients realize the ability to interpret 3D medical images and thus improve doctor-patient communication. Our study has three contributions:

(1) We propose an evidence-based study on communication methods and techniques for doctor-patient communication during interpreting 3D medical images. Also, our study provides rich details on patient learning behaviors, characteristics, and design preferences.
(2) We propose a refence for the design strategies of multimodal medical visualization tools for interpreting 3D medical images.
(3) The application of our design case opens up the possibility of proposing better design considerations in medical settings.

## 2 Background

### 2.1 Tools for interpreting 3D medical images

3D medical image data are obtained from computed tomography (CT), magnetic resonance imaging (MRI), positron emission tomography (PET), and etc. (Gao et al., 2022; Kumar et al., 2013). The application of Artificial Intelligence (AI) in the field of 3D medical imaging has grown rapidly over the past few years, leading to unprecedented breakthroughs in various medical imaging tasks such as image annotation, image interpretation (Teiga et al., 2024), image recognition, and localization of complex patterns (Zhou et al., 2022). Roy et al. (2014) developed a visualization tool for 3D medical image reports that can help physicians and patients easily understand 3D medical images by automatically generating 3D visual summaries. J-Donald et al. (Tournier et al., 2019) developed a lightweight tool for the analysis and visualization of 3D medical images. Their work highlights the need for lightweight tools. Loening and Gambhir (2003) developed a software for multimodal medical image analysis to help physicians interpret and analyze medical images. Their work highlights the functions that can help users interpret and analyze the medical images. To improve the educational efficiency of medical students, Jodi et al. (Crossingham et al., 2009) created a 3D interactive visualization model website to help medical students interpret the anatomy of the liver. McGhee (McGhee, 2010) employed a hybrid approach combining 3D computer-generated imagery (CGI) and clinical MRI data to create a medical visualization tool that offers interpretation services to patients. Similar software such as 3D-Doctor, OsiriX, and 3D Slicer (Pieper et al., 2004) have been maturely used by medical and non-medical platforms for image analysis, visualization, clinical support, etc. (Kikinis et al., 2013). Numerous previous studies have demonstrated that 3D medical visualization can be a useful learning tool (Means et al., 2023). However, more learning tools are developed for professionals with medical backgrounds, and fewer ones are developed for the public and patients. In addition, little attention has been given to study the considerations that influence patients’ interpretation of 3D medical images.

### 2.2 Creating multimodal medical visualization tools

Multimodal medical visualization involves the fusion of datasets from various medical imaging acquisition methods, each capturing different tissue characteristics. This approach can produce multiple visualization modalities. From a medical visualization perspective, it can reduce complexity and cognitive load, improve or accelerate decision support, provide specific applications, etc (Lawonn et al., 2018). Previous studies have shown that visual annotations in medical examination reports are highly beneficial (Fan et al., 2011; Armato, 2003; Reiner and Siegel, 2006). Additionally, embedding educational text within medical images can further enhance understanding of diseases. Simple images are more effective than CT scans in aiding patients’ comprehension (Krasnoryadtseva et al., 2020). For this reason, compared to studies that explore the impact of patients’ understanding on a single type of data or unimodality, relatively few studies have examined the combination of multiple modalities in the context of multidimensional data (such as diagnostic textual data, imaging data, and examination data) within the consultation setting, and the creation of a multimodal medical visualization tool may provide stronger support for patients to break down barriers of specialized knowledge, reduce the cost and timeliness of learning, and promote the effective comprehension of medical information.

## 3 Research process and results

**FIGURE 1:**
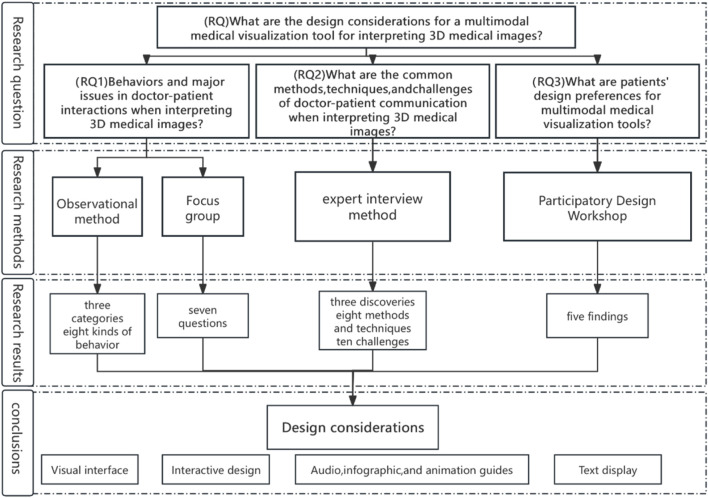
Flowchart design of the study.

We adopted qualitative research methodologies to address the core research questions (Figure 1). (RQ) What are the design considerations for a multimodal medical visualization tool which can interpret 3D medical images? We further divided the core research question into three sub-research questions: (RQ1) the behaviors and main problems of doctor-patient communication when interpreting 3D medical images. First, we used purposive sampling method to randomly select patients for consultation. Those patients were accompanied by us and went to several hospitals in East China. We excluded those cases with invalid consultation (e.g., very short-time consultation), and finally determined eight people as the subjects. The subjects were observed during consultation no less than 30 min in order to assess their interactions and behaviors while interpreting the 3D medical imaging reports. Meanwhile, the physicians’ communication methods and skills were also observed during the consultation. We then recruited and randomly sample 9 patients and asked them to attend the focus group discussion so as to understand the problems and challenges facing them when interpreting the 3D medical images. (RQ2) What are the commonly-used methods, skills and challenges of doctor-patient communication when interpreting the 3D medical imaging data. Second, we conducted 30-min semi-structured interviews with eight specialized physicians experienced in medical imaging, using the expert interview method. These physicians were recruited through our medical service contacts and friends. They were asked questions about the problems, commonly-used methods, skills, and challenges of communicating with patients that they often encountered during their daily consultation. (RQ3) What are the patients’ design preferences for multimodal medical visualization tools? Finally, we gathered patients’ preferences by recruiting 12 interested patients to participate in a design workshop, where they collaborated on creating a medical visualization tool tailored to their needs for interpreting 3D medical images.

Our university’s ethics committee approved our research. We obtained the informed consent forms and verbal agreement from all subjects. They were free to quit the study at any time. We paid 40 dollars as the consultation fees to the physicians and offered household items, phone top-ups, etc. as compensation to the participants.

### 3.1 Consultation observation

#### 3.1.1 Participants

**FIGURE 2:**
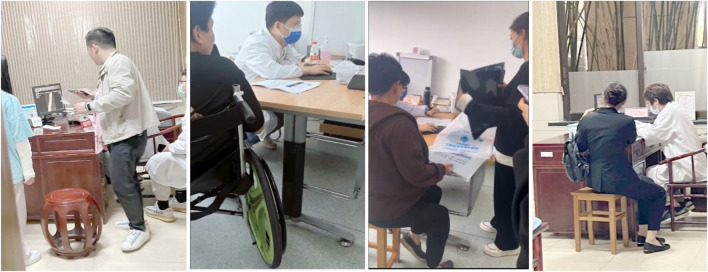
Scenarios of consultation from four groups where the doctors explain the 3D medical images to the patients.

Four doctors and four patients participated in the study (Figure 2). Among the doctors, there are three men and one woman. Two of them are associate chief physicians, and the other two are attending physicians. They all have 5–13 years of medical experience. For the patients, two of them are men, and the other two are women. Their average age is 38-year-old, and their backgrounds are one student, one laborer, one lung cancer patient, and one pregnant woman. Compare to average patients, they have higher requirements and more difficulties in interpreting 3D imaging reports. Among them, two have basic medical skills, three have basic literacy skills, and one have good communication skills. Researchers obtained verbal informed consent from all participants. For detailed demographic information of participants, refer to Supplementay Appendix Table S1a.

#### 3.1.2 Process

We observed four patients from the time they entered the hospital to the end of their consultation (approximately 30 min). Patients mainly used electronic 3D imaging reports for their interpretation, with older patients relying more on physical 3D imaging reports. The consultation process usually took about 10 min, with most of the time being taken by the doctor, who needed to read the 3D medical imaging and then obtained the diagnostic results based on both imaging and textual report. For patients with simple disease, they received the results of the imaging report from the doctor, which belongs to the type of passive acceptance. For patients with more complex disease, the doctor explained the size and location of the lesion in the imaging report, and further used electronic 3D dynamic imaging to verbally explain the evolution of the lesion. We noticed that patients with more complex disease often had anxiety and nervousness, and they paid more attention to the location, changes, and future development of the lesion in the 3D medical imaging, and had more eager knowledge-seeking and communicative behaviours, such as continuing to ask questions about the pathogeny, treatment plans, consequences, etc. By observing the consultation from four groups, it is clear that doctors often used the behaviours of pointing out the location of the lesion by hand (labelling), manually drawing the shape of the lesion (morphology), and metaphorically talking the development of the lesion (metaphors) to help the patients to understand the 3D medical images.

#### 3.1.3 Data collection and analysis

Four research coordinators were responsible for observation, taking notes, and conducting on-site recording and photographing. Before the on-site observation, a senior researcher trained the four research coordinators and developed a detailed observation plan, including the specific observation criteria and annotation guidelines, to ensure that all team members have the same understanding to the research objectives. Once data collection was complete, the research team conducted an internal inspection on the data. Subsequently, the data were systematically analyzed by using the thematic coding (Gibbs, 2007) to ensure the accuracy of the analysis. The data coding process begins by identifying and extracting themes related to the research question from the data so as to identify (RQ1) the patients’ behaviours regarding doctor-patient interactions when interpreting 3D image data in the consultation setting? Each of the four researchers coded the observational data and then checked the results against each other. Then, they negotiated, discussed, and solved the differences among coding to ensure the reliability of the results.

#### 3.1.4 Results

We identified three types of interactions and eight behaviours between patients and doctors when interpreting 3D medical images (Table 1). The main findings are as follows (CO#1-CO#5^1^): Within these findings, we further identified three types of interactions for the doctor-patient interactions, including patient-led interactions, doctor-led interactions, and doctor-patient bilateral interactions. We summarized eight behaviors regarding doctor-patient interactions during consultation, including (a) narration behaviors, (b) Emotional expression behaviors, (c) Operation behaviors, (d) enquiry behaviors, (e) Physical contact behaviors, (f) Reassuring behaviors, (g) mutual concern behaviors, and (h) two-way communication behaviors. (CO#1) During the consultation, we observed that both doctors and patients always used a polite way for enquiry to start interactions with each other. When patients come for a consultation, they usually sit quietly and gaze at the doctor. The doctor will politely ask about their condition, and the patients will describe their symptoms. Some patients (P2/P3) might greet the doctor first, and then directly describe their condition and ask questions to the doctors (a). Notably, when the doctor explains the diagnosis or imaging report results, the patients exhibit high levels of concentration. However, they also show signs of confusion (such as leaning forward to listen intently, asking questions about what they don't understand, or even requesting to view the images together with the doctor). (CO#2) We have observed that most of the patients have physical activities to convey their emotions during the consultation. They want to have a positive interaction with the doctor for their disease, and to learn about the medical knowledge related to their condition. When the doctor interprets the 3D imaging report, P1, P3, and P4 lean forward, stare at the doctor, stand up to point at the imaging report, and ask the doctor questions (b). The doctor operates the patient’s 3D images on the computer (c). Meanwhile, P1 and P4 also view the images on their own phones, point out locations on the images, and ask the doctor for medical information (c). (CO#3) Patients review and are concerned with their imaging together with their physician during the consultation. P1, P3, and P4 show their mobile or printed imaging reports to the doctor to view together. D1, D3, and D4 open the patient’s imaging data on the computer, and use a pen or finger to point out the location of the lesion. They view the images together with the patients while explaining to them (g). P1 and P4 continue to ask related medical questions.

**TABLE 1:** Types and behaviours of doctor-patient interactions.

Types of doctor-patient interaction	Types of behaviors	Examples of behaviors
1.Patient-led interactions	(a) Narration behaviours	Describing the illness
	(b) Emotional expression behaviors	Leaning forward, staring at doctors, finger movements, and interrupting
	(c) Operation behaviors	Viewing images on their own phones, and indicating the location of the lesions
	(d) Enquiry behaviors	Inquiring about the condition and treatment recommendations
2.Doctor-led interactions	(c) Operation behaviors	Spending a long time manipulating and viewing image cases on the computer
	(d) Enquiry behaviors	Polite inquiry with patients
	(e) Physical contact behaviors	Examining and touching the patient’s body
	(f) Reassuring behaviors	Saying “Don’t worry, it’s okay”
3.Doctor-patient bilateral interactions	(g) Mutual concern behaviors	Jointly focusing on the location and size of the lesion and exchanging opinions
	(h)Two-way communication behaviors	Exchanging opinions, and exchanging Question-and-answer

The analysis reveals two methods and techniques that physicians use when interpreting 3D medical images to patients during consultation. (CO#4) Specifying the location of the lesion to the patient is one of the most commonly-used methods. D3 and D4 use a hand or pen to clearly point out the location of the patient’s lesion and verbally describe the changes of the lesion. This helps the patient gain a clear understanding of their condition. (CO#5) Physicians often use metaphors to explain the changes or development of the lesions in 3D medical imaging cases, which is an easy way to help patients understand their conditions. D1 used a tree metaphor to explain an incomplete fracture, describing it as the bark being intact but with cracks inside. D4 compared a calcified lesion to a scar left after skin trauma, explaining the principle that it cannot be restored to its original state.

### 3.2 Patient focus group

#### 3.2.1 Participants

For the focus group, we used public recruitment and snowball sampling to randomly select subjects who are in specific healthcare settings such as clinics, hospitals, etc. In particular, there are 20 people who undergo multiple examination of 3D medical imaging (≧3 times) and thus meet our criterion. According to the personal willingness, nine people were ultimately invited to the focus group discussion. The entire process was recorded without any note-taking on site. We tried to maintain an open dialogue environment and encouraged the patients to share their personal experiences and feelings. Demographic information of the participants is shown in Supplementay Appendix Table S1b. Each participant provided informed consent and received approximately 8 dollars as compensation for their time.

#### 3.2.2 Process

The key objective of the focus group was to explore the problems and challenges patients have in interpreting 3D medical images. The focus group takes 40 min for evaluation by using the COREQ checklist (Gibbs, 2007) and mainly includes three types of questions: 1) patients’ experiences and emotional responses when consultation with 3D medical images, and 2) their ways to understand information and make communication when interpreting 3D medical images. 3) their challenges and expectations for the interpretation of 3D medical images.

#### 3.2.3 Data collection and analysis

In the focus group discussions, each of the two researchers took on a different role: one asked questions to guide the discussion, while the other was responsible for audio recording and observation. The audio recording was subsequently transcribed into textual data. We then analyzed and studied the related texts for theme analysis (Rabiee, 2004). The researchers performed three-level encoding markers for the texts several times, and studied question-related words, phrases, or sentences and use them as the open code. We examined the open code, and grouped them into the spindle code. If no new concepts emerged after multiple encoding, then the theoretical sampling achieves saturation. Based on the results of focus groups, we identified RQ1: the main problems facing the patients when interpreting 3D medical images in the clinical setting. The researchers compared encoding methods by using the Nvivo 20.0 tool to look for differences, and modified the decoding, and finally determined the reliability of the decoding through Kappa coefficients (Table 2).

**TABLE 2:** Patient focus group themes and codes.

Selective decoding	Spindle decoding	Open decoding	Meaning
Experience	1.Emotional experience	1.1Anxiety and confusion	1.Patients’ emotional experiences during 3D medical imaging examinations
		1.2Reassurance and satisfaction	
		1.3Anticipation and exploration	
	2.Ways of receiving images	2.1Pure text diagnosis	2.The ways for the patients to receive 3D medical imaging reports
		2.2Doctor’s interpretation	
		2.3Digital imaging	
	3.Feelings about interpreting images	3.1Simplified interpretation by doctors	3.Patients’ understanding and feelings about doctors’ interpretations of 3D medical images
		3.2Unclear instructions	
		3.3Feeling satisfied and trusting	
Emotional responses	4.Response to the examination	4.1Emotional fluctuations	4.Patients’ emotional responses to repeated 3D medical imaging examinations
		4.2Expectations for the future	
	5.Feelings about the results	5.1Suggestions and hopes	5.Patients’ feelings about the results of repeated 3D medical imaging examinations
		5.2Reliance on doctor and the level of hospitals	
Information comprehension and communication methods	6.Feedback to the doctor	6.1Misunderstanding the interpretation	6.Patients’ feedback on doctors’ interpretations of 3D medical images
		6.2Sufficient Communication	
		6.3Short Interpretation Time	
		6.4 Satisfaction with Interpretation	
	7.Other methods of obtaining information	7.1Obtaining Information Online	7.Patients seeking information from other sources to understand 3D medical imaging results
		7.2 Self-Searching	
		7.3Sharing by Family	
Challenges and expectations	8.Later effects of imaging examination	8.1 Regular Follow-up	8.Patients’ perceptions of the long-term effects of 3D medical imaging examinations
		8.2 Repeat Examinations	
		8.3 Radiation Dose	
	9.Opinions	9.1 Information and Data Sharing	9.Patients’ expectations or hopes for future 3D medical imaging
		9.2 Lightweight and Fun	
		9.3 Radiation Safety	
		9.4 Equipment Improvement	
		9.5 Intelligent Interpretation	

#### 3.2.4 Results

We identified seven primary issues that patients face when interpreting 3D medical images (FG#1-FG#7^2^). (FG#1) Patients’ emotional reactions during 3D medical imaging examinations are usually triggered by uncertainty about the examination process and results. However, timely interactions with medical staff could improve the sense of reassurance and satisfaction of some patients. The patients’ expectation and exploration of the results reflect their curiosity and concern for their health. (FG#2) Patients have different selection preferences when receiving 3D medical images. Some patients prefer pure textual diagnosis, some prefer digital images, and most patients prefer to listen to the explanation of their doctors. (FG#3) The patients’ understanding and feel of 3D medical imaging results depend on the sufficiency of interpretation of the doctors. Doctors’ failure to explain diagnostic results may lead to patient misunderstanding, and thus make them get confused, and consequently lead to worry or dissatisfaction with the physicians’ interpretation of the results. (FG # 4) Patients will trust and reply more on the examination equipment, doctors and hospitals after they undergoing multiple 3D medical imaging examinations. They expect a high level of professional competence from the doctors and hospitals, and hope to get timely and accurate interpretation and treatment plans. This suggests that the level of trust and reliance on the doctors and hospitals has a significant impact on the patients’ satisfaction and emotional reactions. (FG#5) When interpreting 3D medical images, patients usually take three ways to understand the information: direct communication with the physician, patient self-understanding, and information obtained from other sources. Specifically, most patients rely on the physician’s interpretation to understand the imaging results. However, some patients may get confused by the physician’s interpretation, and will seek additional information or help from other sources to enhance their understanding. Patients will use methods such as self-study, sharing information from friends and family, or internet searches to improve their understanding and communication. Nonetheless, those patients who received adequate communication from their physicians were usually satisfied with the interpretation results. This variety of ways to access information demonstrates the different needs and responses of patients when facing 3D medical images. It highlights the importance of the physicians in providing clear, comprehensive interpretations.

In addition, (FG#6) Periodic review and repeat examinations were frequently mentioned in terms of the post-effect of the imaging examinations. This may indicate patients’ needs for disease tracking and management, and also reflects the importance of medical imaging in disease management and treatment. Furthermore, concerns about issues such as radiation dose and the limitations of cross-modality translation suggest that patients are concerned with the safety of imaging examinations and cross-modality translation techniques. (FG#7) Patients have various expectations of the healthcare services in the field of medical imaging. This includes in-depth information sharing so that patients can have a more comprehensive understanding of their health and treatment plans. Patients expect healthcare providers to utilize advanced technology to enhance the intelligence of interpreting imaging reports, thereby improving the accuracy and efficiency of interpretation. In addition, patients have clear expectations for the radiation safety, and technological advancements in medical equipment (including the lightweight and fun designs for equipment). These expectations reflect patients’ concerns and needs for improvements and technological developments in medical imaging services.

### 3.3 Semi-structured interviews with physician experts

#### 3.3.1 Participants

We interviewed eight specialized physicians through our medical service network contacts and friends. We conducted one-on-one semi-structured interviews with the eight physician specialists. Among them, three are females and five are males. Their average age is 43-year-old. All of them often need to interpret 3D medical imaging reports to the patients during their consultation. The affiliation of the eight doctors ranges from tertiary hospitals in first-tier cities to local primary-level hospitals. The eight doctors all have more than 8 years of medical qualifications (Supplementay Appendix Table S1c).

#### 3.3.2 Process

The purpose of this interview is to understand the common methods, techniques, and challenges of doctor-patient communication when interpreting 3D medical images? We conducted eight separate unstructured interviews with physician experts, each lasting less than approximately 30 min. We enforced uniform standards for the COREQ checklist (Gibbs, 2007) and asked the experts two main questions: 1) When interpreting 3D medical images (CT\MRT, etc.) to patients, what are their commonly-used methods or techniques used to help the patients understand and learn about their disease? 2) What are the challenges or suggestions if designing a software tool that can help patients understand 3D medical images? The interviews were conducted using a combination of offline and online ways, and we also asked the physician specialists to share any interesting things that happened during the consultation.

#### 3.3.3 Data collection and analysis

In the interviews, two researchers interviewed one physician expert. One researcher was responsible for asking questions according to the outline of the interview, while the other was responsible for audio recording and taking notes on the interview highlights. After each expert interview, we transcribed the audio data into the textual data, and conducted the thematic analysis by analyzing the text related to the research questions (Rabiee, 2004). Also, in the semi-structured interviews we focused on analyzing the methods and techniques used by the doctors when helping patients to understand 3D medical images. The researchers performed conceptual and categorical decoding on the in-depth interview data of 8 doctors through multiple times content analysis, and finally determined 22 open decoding and 3 spindle decoding (Table 3).

**TABLE 3:** Themes and codes from physician interviews.

Spindle decoding	Open decoding
Communication Methods and Techniques	Drawing to Make Things Concrete
	Reassuring Patients’ Psychology sentiments
	Clear and Simple Explanations
	Image Description
	Comparison with Normal References
	Analogies and Examples
	Indication the location
	Attention to Predict the Consequence of illness
Workflow of Consultation Behaviors	Condition Diagnosis
	Condition Examination and Testing
	Condition Education and Popularization
	Treatment Considerations
Challenges and Suggestions	Multimodal Design
	Patients Lacking Medical Knowledge
	Complex Patient Needs
	3D Medical Image Reconstruction
	Collecting Information from Online Big Data
	Alleviating Concerns and Boosting Confidence
	Sharing Medical Information Resources
	Limited Medical Resources
	Adding Comparison Images
	Adding Medical Animations for Educational Purposes

#### 3.3.4 Results

This study revealed three findings (SI1-SI3): (SI#1^3^) Doctors usually follow a fixed flow of consultation when interpreting 3D medical imaging results: “Examination-Diagnosis-popularizing knowledge-Treatment”, and expect patients to have a high level of compliance. The interviewed doctors believe that “patients lack medical knowledge and thus have difficulties in understanding medical imaging,” “different patients with different diseases have different complex needs,” “the most important thing to explain the condition to the patients is to make an accurate diagnosis,” “do some simple pathophysiological explanations,” “According to the basic examination, we come to the conclusion that what is the nature of the disease, and whether it is necessary to perform other examinations,” “patients need to dispel doubts and enhance confidence in the limited medical resources and treatment time,” (SI#2) The commonly-used methods and techniques used by doctors when interpreting 3D medical images to the patients mainly include: indicating the location, describing the Images, drawing to make things concrete, soothing the patients’ psychology and emotion, making the explanations easy to understand, illustration, metaphor, taking examples, comparing with references, using prognosis, etc., to help the patients understand and know about their conditions. The interviewed physicians mentioned the following: “I roughly indicate the location of the disease for the patients,” “I usually use drawings to explain which part is abnormal,” “I typically describe what I see in the images when interpreting the imaging to the patients,” “Sometimes, I use models or normal images to help patients understand more visually,” “explaining the condition to the patients in easy-to-understand ways through examples from our life or simple metaphors to make them have a better understanding,” “Be patient when talking and don’t rush. The patients’ emotions rely on the doctors’ attitude,” “We need to make judgments beforehand and have an adequate communication with the patients.” (SI#3) 3D medical image reconstruction technique, animation and popularization of medical knowledge, multimodal design and display, and big data support can help both doctors and patients gain a clearer and more accurate cognitive understanding. However, it is still necessary to include the 2D data and other medical indications. Doctors prefer to collect as much medical evidence as possible to make a qualitative diagnosis. The interviewed physicians suggested the following: “It would be great to have both images and text, with interspersed images and 3D models, to quickly help patients understand where the issue is and make accurate judgments about the disease,” “3D reconstruction is relatively intuitive,” “If patients have doubts about their examination results or lack knowledge, they can refer to big data to find relevant information,” “if we can popularize the knowledge by using medical animation, then patients may be able to understand it by themselves”.

### 3.4 Patient participatory design workshop

#### 3.4.1 Participants

Through community platforms and university campuses, we recruited 12 patients who need to regularly or multiple times (≧3) undergo medical imaging examination and asked them to participate in our participatory design workshop. Among them, 4 are males and 8 are females, as shown in Supplementay Appendix Table S1d. Three of our researchers participated in the design workshop, with one researcher chairing the meeting and two researchers documenting and helping patients complete the workshop design tasks. We provided the electronic informed consent for each participant and obtained their verbal agreement. In addition, we give each participant approximately 13.8 dollars as their compensation for topping up.

#### 3.4.2 Process

**FIGURE 3:**
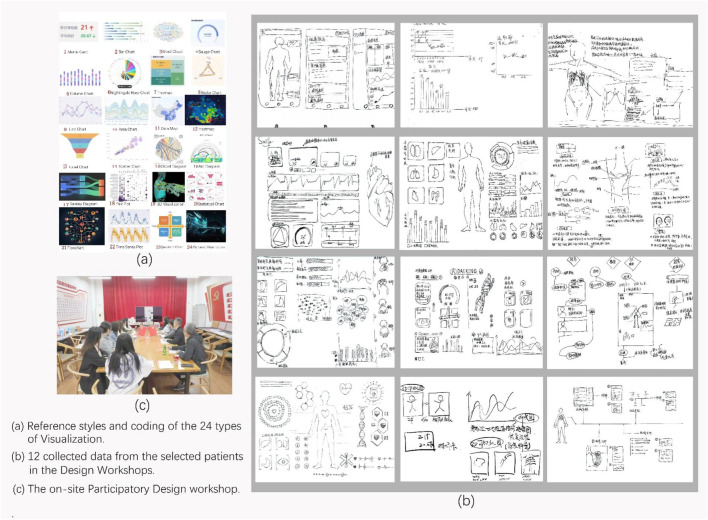
Materials from the Patient Participatory Design Workshop. **(a)** reference styles and coding of the 24 Types of Visualization. **(b)** 12 collected data from the selected patients in the Design Workshops. **(c)** the on-site Participatory Design Workshop.

The goal of the design workshop is to investigate the patients’ design preferences for the multimodal visualization tool which can be used to interpret 3D medical images, e.g., what elements, presentation, colors, and functions (visual appearance, images, text, voice, sound, motion, animation, and interaction design factors) to use. The workshop task involved having 12 patients assemble their ideal medical visualization tool within 60 min by using the visualization materials provided by us. These visualization materials are designed to support the understanding of medical data. Moreover, the patients need to label the expected functions for their ideal medical visualization tool. In case the participants do not know how to get started, we provided them with 24 types of visualization materials as reference. These materials were presented using both electronic and paper cases. We also encoded these visualization materials (labeled from 1 to 24) to help the patients effectively relate to the actual medical data (Figure 3).

#### 3.4.3 Data collection and analysis

We collected 12 visualization tools completed by the patients, and performed analysis on the collected data. We analyzed the visualization, elements, presentation and functions corresponding to the data (Gameiro et al., 2018). Three researchers participated in data collection and analysis.

#### 3.4.4 Results

We analyzed and summarized five key findings from the collected data (DW#1-DW#5^4^). (DW#1) We found that for the organ-related medical data, participants prefer to use 3D real-time interactive visualization to show them. (DW#2) 3D visualization, dynamic visualization, statistical charts, and measurement charts are the most popular medical visualization methods chosen by the participants, which reflects the participants’ preference over visualization methods. (DW#3) Participants are more concerned about the combination of functions of different modules, and not so much about the colors of the tools. Some patients prefer a simple solid-colored background, and this may be related to the fact that participants are not good at color matching. (DW#4) Participants expect voice introduction and word cloud maps when visualizing text data. (DW#5) Participants prefer a combination of images and text for case-related medical knowledge, such as choosing knowledge graph visualization methods for presentation.

## 4 Design considerations

By summarizing and analyzing the results of our four empirical studies and previous research, we propose seven design considerations for a multimodal medical visualization tool that can be used to interpret 3D medical images. The design considerations are based on four aspects: the effect of visual interface, interaction design, medical education and popularization, and text presentation.

### 4.1 Effect of visual interface

1. Previous studies have shown that both simple and complex medical visualization tools are significant for the patients to improve their understanding of medical knowledge (Mahmoudi et al., 2010). Simple and varied infographics bring easy-to-grasp medical knowledge and enhance decision-making for patients (McCrorie et al., 2016), and complex visualization can enhance learning engagement (Dankbaar MSc, 2015) and effectiveness (Cook et al., 2008). According to our empirical studies, patients prefer 3D visualization tools to demonstrate organ images as well as location and size of the lesions. (DW#1\SI#3) Compared to the uncertainty of 2D slices, spatial anatomy-based 3D medical images are more highly cognitive and deterministic.
2. While key 2D slices have the basic diagnostic information that doctors can trust, 3D medical images can lower the barrier to understand professional medical knowledge. (DW#1\SI#3) The real-time and synchronized comparison display of 2D and 3D medical images can further improve the cognitive consensus between doctors and patients.

### 4.2 Interaction design plan

3. The physicians need to clearly demonstrate the development of the lesion, its current status, and its intervention methods to the patients, e.g., its shape, size, location, cause, and consequence (SI#2). Therefore, the interaction design plan of the visualization tool needs to show the complete shape, size and location of the lesion based on the simple interaction with the 3D medical image model. This requires the separation of the organ tissues (blood vessels, etc.) and the lesion tissues visually, and distinguishing the normal features from the abnormal ones. For example, by using commonly-used interaction techniques such as manually marking different colors, setting the transparencies of different tissues, manually drawing lines, comparing with references, etc., it is easier for the doctors and patients to understand.
4. From the location perspective, both doctors and patients need to know the specific location of the lesion and its relation with the surrounding normal tissues, and this can help doctors observe and explain the treatment plans. Therefore, the interaction design plan should include the function of 360-degree rotation.

### 4.3 Audios, infographics, and animation guides

5. Doctors often need to explain specialized medical knowledge when interpreting medical images (SI#3). However, since the patients lack the knowledge, there will be miscommunication between doctors and patients. Based on the learning tools, medical education and popularization can significantly improve patients’ understanding and perception of medical knowledge. Videos and animations can bring more medical knowledge and learning effectiveness (Knapp et al., 2022), while infographics are increasingly used in medical education and found to be more efficient (Peters and Nordness, 2023). Taking advantage of audios, infographics, and animation guides can help patients quickly understand the causes and consequences of their condition. Therefore, we should include the case-related audios, infographics, and animation guides into the design considerations.

### 4.4 Text presentation

6. Text data is the most direct diagnosis from the imaging examination, and the patients often lack knowledge to the text diagnosis and thus need to learn the relevant medical knowledge (FG#5). Design considerations can include hyperlinks to the keywords to help patients better understand the content of the diagnostic results.
7. Most patients, such as the older adults, disabled, illiterate, etc., have cognitive difficulties with simple text data because of their level of vision, cognitive level, educational level, etc. Therefore, the addition of synchronized audio interpretation can break through the barriers of understanding.

## 5 A case study of the design of a multimodal medical visualization tool

There are seven basic design considerations a multimodal medical visualization tool should have for interpreting 3D medical images. These design considerations are from four aspects: visualization interface, interaction design, education and popularization, and text presentation.

1. Employing 3D reconstruction of real medical images for visualizing organs, tissues, and lesions.
2. Synchronously displaying 2D images and 3D reconstructed visualization in the same interface side by side;
3. Capability to separate normal and abnormal organs or tissues. For example, you can manually mark different tissues with different colors and transparency, or manually draw lines around certain organ/tissue and compare it with the reference object.
4. The 3D visualization of real medical images should be able to rotate (within the range of 360°), translate, zoom in and zoom out.
5. Medical cases should be accompanied by education and popularization explanations, such as audio, knowledge graphs, infographics, and animated guides explaining etiology and intervention plans relevant to those medical cases.
6. There should be textual diagnostic results in the imaging report., Also, it is necessary to add hyperlinks to the key words regarding each medical case so as to provide correct theoretical support.
7. Providing synchronized audio interpretation for text results and query results.

### 5.1 The weight analysis of design considerations

This research asks the advice of experts for the form of developing the visualization tool (web application, software, VR/VR platform). To improve the reliability of the weights of design considerations, the expert panel consists of 4 clinicians (they are from departments of cardiovascular, orthopedics, and radiology), 1 medical education expert, and 3 human-computer interaction designers. Among the panel, 5 experts have more than 10 years of experience in doctor-patient communication. Meanwhile, considering that the weights can reflect the needs and cognitive abilities of different patients, 12 patients were invited to score the design considerations (1–9 scale method). We divided patients into three levels (low/medium/high) according to their medical literacy, and logically screened them through conditions such as age and education background to achieve the differentiation of their medical literacy. The expert panel was divided into four groups according to the fields and medical literacy, and each group of experts make the score by using the weighted evaluation method to improve the objectivity of analysis. We construct the judgement matrix and weights of the design considerations based on the Analytic Hierarchy Process (AHP), as illustrated in Table 4.

**TABLE 4:** The summarized table of factors of the design considerations.

A	$\lambda_{\max}$	$\omega_{i}$ (%)	CR	B	$\omega_{i}$			CR
					WEB	Software	VR\AR	
A1 visual interface	4.088	25.352	0.033	B11 3D visualization	0.109	0.582	0.309	0.004
				B12 2D/3D synchronized comparison	0.135	0.784	0.081	0.033
A2 interaction design	4.088	57.091	0.033	B21 tissue feature separation	0.089	0.588	0.323	0.009
				B22 the 360-degree rotation	0.082	0.315	0.603	0.002
A3 medical education and popularization	4.088	12.766	0.033	B31 audio	0.143	0.571	0.286	0.000
				B32 infographics	0.23	0.648	0.122	0.004
				B33 animation	0.109	0.309	0.582	0.004
A4 text presentation	4.088	4.791	0.033	B41 the keyword hyperlinks	0.74	0.167	0.094	0.013
				B42 synchronized audio interpretation	0.117	0.614	0.268	0.017

The results show that the design consideration factors (A primary factors) that have the relatively big weights are interaction design (57.091%) and visual interface (25.352%), and the ones that have relatively small weights are text presentation (4.791%), and medical education and popularization (12.766%). As a result ( $\lambda_{\max}$ = 4.088, RI = 0.882, CI = 0.029), CR = CI/RI = 0.033 < 0.1, it passes the consistency test.

Specifically, the weights of factors of the design considerations (B primary factors) are different in the web application, software and VR/AR platforms. For example, the keyword hyperlinks (0.74) have a significant advantage in the web application. Conversely, their significance is dramatically reduced (0.094) in the VR/AR technique. 2D/3D synchronized comparison (0.784) and infographics (0.648) have relatively big weights in the software, which indicates that when developing software, these two visualization techniques can more intuitively and efficiently present the complex data or scenarios, and meet the needs of users to obtain rich details of information. For VR/AR technique, the 360-degree rotation (0.603) has relatively big weights, which indicates that it allows to obtain a better immersive experience from an all-round perspective. All design consideration factors’ CR < 0.1, and pass the consistency test. Therefore, we will follow the weights of the design consideration factors to develop a multimodal medical visualization application.

### 5.2 Multimodal medical visualization tool for supporting doctor-patient communication

**FIGURE 4:**
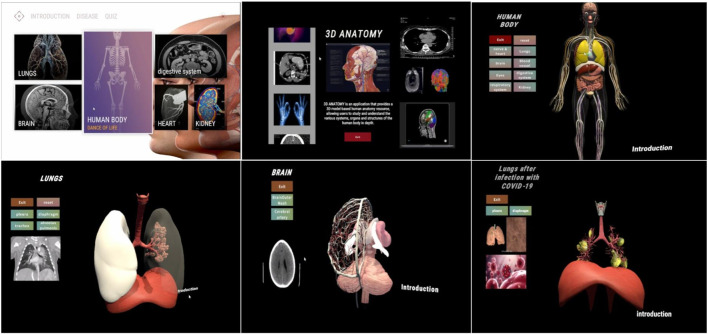
Demonstration of the interface of the multimodal medical visualization tool.

According to the design considerations, we completed a multimodal medical visualization tool based on the modeling of real 3D medical images data to assist doctor-patient communication in medical context (Figure 4). From the perspective of generalized design, this tool is designed to meet the demand of patients with different levels who want to interpret and understand 3D medical images.

The visualization tool comprises three interactive steps. (a)The first step is to input the patient’s information to log into the account. (b)The second step is to select a completed imaging report or upload local imaging data and click generate. (c) Synchronously displaying both two-dimensional and three-dimensional data. A text button allows the user to select which tissue to be displayed. The scroll wheel is used to set the transparency of the visualization, while the palette buttons are used to adjust the colour of the tissue. The pen button allows the user to draw lines or writing words. The left button of mouse allows to rotate the visualization, the right button of mouse allows to translate the visualization, and the middle button of mouse allows to zoom in or zoom out the visualization. Clicking the sound button can play an audio introduction, while clicking the video button can open a pop-up window containing videos of medical knowledge. Finally, clicking on the keywords hyperlinks to related websites.

The developed design tool has five main functions: 1) 3D reconstruction of real data, 2) in-depth learning of human anatomical structures, 3) multimodal visualization, 4) guidance for medical education and popularization, and 5) inter-modality translation using artificial intelligence.

### 5.3 Technique scheme

The 1.0 version of this system is developed based on Unity3D, and it aims to provide a visualization tool that can interact with human anatomy. The tool contains a variety of human anatomical models and lesion models, and utilizes C# scripting to implement various interaction functions, such as translation, zooming out/in, rotation, and visibility adjustment of the models. With the UI components in Unity, an intuitive and friendly user interface was designed, including elements such as control panels, menus and buttons. The interface is designed to adapt to users with different levels of digital literacy. The responsive layout of the interface is adapted to 4–27-inch screens (from mobile screen to large screen). The core interactive technique supports both mouse and touch-screen gestures, and the torch-screen gestures include panning, zooming in/out, and rotating. In addition, the visualization tool contains a rich introduction to anatomical knowledge, which is presented to the user in the form of text, images, audio and animation. Moreover, in terms of its scalability and future adaptability, its existing architecture has reserved an expansion interface, and its modular design supports progressive technology iteration (e.g., VR/AR visualization plug-ins, and Unity/Unreal platforms). In terms of software upgrades, it can integrate GAN-based segmentation models so as to realize the modality translation and pre-processing of the medical images.

### 5.4 User evaluation of the prototype

#### 5.4.1 Research methods and process

The research uses a combination of Analytic Hierarchy Process (AHP) and Fuzzy Comprehensive Evaluation (FCE), that is, Fuzzy Analytic Hierarchy Process (FAHP). Quantitative data relevant to the evaluation of our prototype were collected by means of questionnaires. The main steps of the study are to construct user evaluation metrics using the AHP method, and to construct the judgment matrix and weight allocation step by step, and then to use the FCE method to obtain the comprehensive evaluation of the prototype from the users so as to initially measure the effectiveness of the tool in the clinical setting.

The evaluation objective of the study is the patients’ evaluation of the visualization tool prototype. According to the TAM technology acceptance model and relevant literature, the first-level metrics of evaluation include three evaluation dimensions, which are perception, cognition, and behaviours. The second-level metrics of evaluation include a total of seven evaluation metrics, which are perception usefulness (Davis, 1987), perception ease of use (Davis, 1987), cognitive relevance (Chen et al., 2022), cognitive comprehension (Dorosh et al., 2013), cognitive memory (Williams and Drew, 2019), behavioral communication skills (Lee et al., 2002), and behavioral decision-making skills (Dimara et al., 2017). Then, the FCE method is used to construct the metric set, construct the membership matrix, determine the weight vector of the factor set, and finally compute the comprehensive evaluation vector and comprehensive evaluation scores.

#### 5.4.2 Participants

The AHP method uses an expert panel scoring method. We consulted with 5 physician experts and 3 development engineers in the expert panel. 12 patients who had undergo medical imaging examinations for a long time (who have differences in medical literacy) scored the judgment matrix of each metric (1-9 scale method).

In the FCE method, we recruited the outpatient cases on the site to perform initial small-sample trial of user evaluation. We demonstrated the prototype of the tool and give the electronic questionnaire to 64 outpatient patients in the hospital. Participants were divided into 6 age groups (11–60 years old) according to 9 years intervals, and they have diverse digital literacy. Invalid questionnaires such as filling the answers in a very short time, filling the same answers, or quitting midway were manually excluded, and thus 41 valid questionnaires were obtained. The questionnaires use a five-point Likert scale, and outpatient patients make scores according to the degree of evaluation metrics. We manually count the number of participants in each scale of each metric, and thus obtain the quantitative scores of the user evaluation.

#### 5.4.3 Data collection and analysis

According to the square root method, the AHP weight analysis applies the AHP equations to the four judgment matrices, respectively, to obtain the summary results of weight analysis of each metric (as illustrated in Table 5). The results show that in the judgement matrix, the perception weight of A1 is about 0.081, and the cognitive weight of A2 is about 0.188, and the behavior weight of A3 is about 0.731. In the judgment matrix of A2 cognition, its results are: ( $\lambda_{\max}$ = 3.065, RI = 0.525, CI = 0.032), CR = CI/RI = 0.06 < 0.1, and thus it passes the consistency test. According to the weight values of each second-level metric, the global weights Wu = (0.061, 0.020, 0.125, 0.045, 0.018, 0.487, 0.244)^T^ are obtained.

**TABLE 5:** Summary of AHP (Analytic Hierarchy Process) results for each metric.

Metric	${A\omega }_{i}$	$\omega_{i}$ (%)	$\lambda_{\max}$	CI	RI	CR	Local weights	Global weights
A1 perception	0.362	8.096	3.065	0.032	0.525	0.062	0.08	0.081
A2 cognition	0.843	18.839					0.19	0.188
A3 behaviour	3.271	73.064					0.73	0.731
B11 usefulness	1.501	75.01	2	0	0	0	0.75	0.061
B12 ease of use	0.499	24.99					0.25	0.020
B21 relevance	2.696	66.6	3.076	0.038	0.525	0.073	0.666	0.125
B22 comprehension	0.969	23.942					0.239	0.045
B23 memory	0.383	9.458					0.095	0.018
B31 communication	1.333	66.667	2	0	0	0	0.666	0.487
B32 decision-making	0.666	33.333					0.333	0.244

According to the evaluation objectives, the FCE evaluation analysis establishes a factor set U = {usefulness, ease of use, relevance, comprehension, memory, communication ability, decision-making ability}. Then it establishes a rating set V = {low, medium low, medium, medium high, high} that is relevant to the rating grade. According to the membership formula of each evaluation factor to the evaluation grade, we can obtain R, and so on to obtain R1, R2, R3. For example, the overall fuzzy evaluation matrix R is as follows:

$$R=\left[\begin{array}{c} \begin{array}{cc} 0.000 & 0.171 \end{array}\;\begin{array}{ccc} 0.268 & 0.415 & \;0.146 \end{array}\\ \begin{array}{cc} \;0.024 & 0.122 \end{array}\;\begin{array}{ccc} 0.317 & 0.342 & \;0.195 \end{array}\\ \begin{array}{cc} \;0.000 & 0.171 \end{array}\;\begin{array}{ccc} 0.293 & 0.488 & \;0.049 \end{array}\\ \begin{array}{cc} \;0.000 & 0.122 \end{array}\;\begin{array}{ccc} 0.415 & \;0.268 & 0.195 \end{array}\\ \begin{array}{cc} 0.073 & 0.244 \end{array}\;\begin{array}{ccc} 0.415 & \;0.195 & 0.073 \end{array}\\ \begin{array}{cc} 0.024 & 0.073 \end{array}\;\begin{array}{ccc} 0.317 & \;0.415 & \;0.171 \end{array}\\ \begin{array}{cc} 0.049 & 0.220 \end{array}\;\begin{array}{ccc} 0.390 & \;0.220 & \;0.122 \end{array} \end{array}\right]$$

According to the importance of the evaluation factors, we determine the weight of each factor. The weight vector satisfies the condition $\sum_{i=1}^{n}a_{i}=1$ and $a_{i}$ ≥0. According to the relevant formula and the weight vectors of the factor set in the AHP method Wu and W1 = (0.75,0.25), we can obtain the weight vectors of the rating set Wᵛ = {0.067,0.133,0.200,0.267,0.333}. According to the formula B = W ∘ R, we can obtain the comprehensive membership, and the overall metric’s B = {0.025,0.133,0.335,0.365,0.142}; B1 = {0.006,0.159,0.280,0.397,0.158}; B2 = {0.007,0.166,0.334,0.408,0.086}; B3 = {0.032,0.122,0.341,0.350,0.155}. The final evaluation results are determined according to the methods such as the dual-weight formula.

#### 5.4.4 Results

The comprehensive rating value was obtained by the dual-weight method μ ≈0.231, and we check the weight of the rating set Wᵛ. Therefore, the comprehensive raging value ranges from medium to high. The comprehensive membership of the overall metric is B = {0.025, 0.133, 0.335, 0.365, 0.142}. According to the principle of maximum membership, the maximum membership value of the rating of the users to the prototype is 0.365. According to the evaluation level, the overall evaluation corresponds to a relatively high level. The maximum membership value of perception B1 is 0.397, the maximum membership value of cognition B2 is 0.408, the maximum membership value of behaviour B30s 0.350, and they are all at a high level. Therefore, the user evaluation is quantitatively analyzed from 7 evaluation metrics in the three dimensions of perception, cognition, and behaviour, and the results showed that the prototype performs well in these aspects. We plan to validate the visualization tool in the clinical setting in the future.

## 6 Discussion

Our empirical study explores the benefits of designing a multimodal medical visualization tool for patients to interpret 3D medical images. While most of the previous studies focused on the impact of a particular visualization method or technique on both doctors and patients, this study explores the benefits of combining multiple visualization methods for both doctors and patients. It also expects to broaden the application of visualization techniques in medical domain and provide more comprehensive evidence to support effectiveness and superiority. The multimodal form of combining multiple visualization methods can compensate for the deficiencies and limitations of a single method, provide abundant and multidimensional display of medical information, and make it easier for patients with different cognitive levels to understand complex medical knowledge. Through systematic research, our study can provide evidence support for future clinical practice, and provide theoretical guidance and reference for the development of future medical visualization tools.

There are still some challenges multimodal visualization design tools facing when interpreting 3D medical images. First, the accuracy of reconstructing all tissues in the data, which may not be applicable in in all circumstances. Second, whether the reconstructed details conform to the real anatomy, and can be used to support diagnosis and treatment plans effectively. Also, whether they can be used in conjunction with virtual simulation techniques to demonstrate operation plans. While there are significant advancements, further application and integration into clinical practice remain a challenge.

## 7 Limitations

This study has two limitations. Firstly, the relatively limited sample size of our empirical study may have an impact on the extent to which the results can be generalized. Our empirical studies have a limited sample size and geographical limitations, which may affect the generalization of the findings. Therefore, in the future we plan to conduct a multi-center validation study with the cooperated hospitals. Nonetheless, we derived generalized design strategies from our empirical results and previous studies in order to enhance the applicability of our findings. Secondly, the development of multimodal design tools cannot be limited to the current experimental setting and conditions. It may also be necessary to consider additional factors, including different medical background, socio-cultural relationships, and technological advancements and constraints. Therefore, we design a multimodal visualization tool to demonstrate how design considerations can be applied to a specific context. In the future work, we hope to evaluate its usability, effectiveness and impact in the real-world clinical settings. Also, in terms of its scalability and future adaptability, its existing architecture has reserved an extension interface, and its modular design supports progressive technology iterations (e.g., VR/AR visualization plug-ins, Unity/Unreal platforms).

## 8 Conclusion and future work

The research aimed to investigate patient interactions and behaviours when interpreting 3D medical images, to observe physician communication methods and techniques, to learn about the issues, common methods, skills and challenges during doctor-patient communication, and to identify the main problems encountered by patients when interpreting 3D medical images and find out the design preferences of the problem-solving tools. Based on these findings, we provide design considerations for visualization tool from the following four aspects: visual interface effect, interactive design, medical education and popularization, and text presentation.

For future work, we need to employ evaluation metrics to improve prototype based on the experts’ evaluation, perform test on a large number of patients by using quantitative analysis, and refine the prototype and design strategy. Additionally, we will explore the implementation of different multimodal visualization methods so as to improve patients’ cognition, attention, and interests, etc. Furthermore, we plan to use the visualization tool to perform 200 RCT studies in order to understand its role in patients’ emotional impact, improved comprehension ability, doctor-patient interaction time, decision support, etc.

## Data Availability

Due to medical privacy and ethical considerations, the datasets generated and analyzed during this study are not publicly available. However, they can be obtained from the corresponding author upon reasonable request.

## Footnotes

1 CO# is the abbreviation of Consultation Observation.

2 FG# is the abbreviation of Focus Group.

3 SI# is the abbreviation of Semi-structured Interviews with physician experts.

4 DW# is the abbreviation of Design Workshop.

## Abbreviations

CO, consultation observation; D, doctor; DW, design workshop; FG, focus group; P, patient; RQ, research question; SI, semi-structured interviews with physician experts.
