# Supplementary material for: Qualitative studies: designing a multimodal medical visualization tool for helping patients interpret 3D medical images
Source: Front Physiol. 2025 May 26;16:1559801. doi: 10.3389/fphys.2025.1559801 (PMC12146198; doi:10.3389/fphys.2025.1559801)
Supplement: Supplementary file 1 [file Supplementaryfile1.docx]

# Supplementary Material

## Appendix 1

Table a. Demographics during consultation observation.

| Identity number | Gender | Age | Characteristics |
| --- | --- | --- | --- |
| D1 | Male | 50 | Orthopedics department |
| D2 | Male | 29 | General surgery department |
| D3 | Male | 32 | Cardiothoracic surgery department |
| D4 | Female | 43 | Gynecology and obstetrics department |
| P1 | Female | 16 | High school student |
| P2 | Male | 39 | Laborer |
| P3 | Female | 57 | Cancer patient |
| P4 | Female | 41 | Pregnant women |

Table b. Demographics of participants in focus group.

| Number | Gender | Age | Summary of Experience |
| --- | --- | --- | --- |
| Participant 1 | Male | 35 | Regular CT review of lung nodules |
| Participant 2 | Female | 52 | Multiple review CTs for back injuries caused by accidents |
| Participant 3 | Female | 40 | Repeat CT for pneumonia with multiple infections |
| Participant 4 | Male | 18 | Multiple imaging of left ankle fracture |
| Participant 5 | Male | 41 | Congenital Heart Disease Multiple Imaging |
| Participant 6 | Female | 26 | Multiple imaging of teeth |
| Participant 7 | Female | 30 | Regular imaging of the person and his/her family |
| Participant 8 | Male | 21 | Regular imaging of the person and his/her family |
| Participant 9 | Male | 16 | Multiple periodic checkups for fractures |

Table c. Demographics of interviewed physicians.

| Interviewed Physicians | Gender | Age | Hospitals | Departments |
| --- | --- | --- | --- | --- |
| D1 | Male | 38 | Hangzhou Honghui Hospital | General surgery |
| D2 | Male | 42 | Guangdong Huidong Hospital | Orthopedics |
| D3 | Male | 51 | Quzhou Second People's Hospital | Respiratory medicine |
| D4 | Female | 46 | Weinan Orthopedic Hospital | Imaging |
| D5 | Female | 43 | Shandong Wujing Hospital | Internal medicine |
| D6 | Male | 50 | Shanghai Xinhua Hospital | Cardiovascular medicine |
| D7 | Female | 41 | Zhejiang University Second Hospital | Neurology |
| D8 | Male | 34 | Hangzhou Hospital of Traditional Chinese Medicine | Integrated Chinese and Western medicine |

Table d. Demographics of Participants in Workshop.

| Number | Gender | Age | Occupation |
| --- | --- | --- | --- |
| Participant 1 | Female | 51 | Cleaner |
| Participant 2 | Female | 19 | University student |
| Participant 3 | Female | 38 | Self-employment |
| Participant 4 | Male | 34 | Teacher |
| Participant 5 | Female | 31 | Administrative staff |
| Participant 6 | Female | 24 | University student |
| Participant 7 | Male | 39 | Postgraduate |
| Participant 8 | Female | 25 | postgraduate student |
| Participant 9 | Male | 43 | Estate management |
| Participant 10 | Female | 21 | University student |
| Participant 11 | Male | 23 | University student |
| Participant 12 | Female | 29 | Receptionist |

## Appendix - COREQ checklist

The Consolidated Criteria for Reporting Qualitative Studies (COREQ): 32-item checklist (Table developed from Tong et al., 2007)

| No. Item | Guide questions/description | Page Number |
| --- | --- | --- |
| Domain 1: Research team and reﬂexivity | | |
| The research team | | 4，5 |
|  | | |
| Personal  Characteristics | | |
| 1. Inter  viewer/facilitator | Which author/s conducted the interview or focus group? | 5，7 |
| 2. Credentials | What were the researcher’s credentials? E.g. PhD, MD | 1 |
| 3. Occupation | What was their occupation at the time of the study? | 6 |
| 4. Gender | Was the researcher male or female? | 7 |
| 5. Experience and training | What experience or training did the researcher(s) have? | 7 |
| Relationship with participants | | |
| 6. Relationship established | Was a relationship established prior to study commencement? | 7 |
| 7. Participant  knowledge of the interviewer | What did the participants know about the researcher e.g. personal goals, reasons for doing the research? | 7 |
| 8. Interviewer characteristics | What characteristics were reported about the interviewer/facilitator e.g. Bias, assumptions, reasons and interests in the research topic? | 7 |
| Domain 2: Study design | | |
| Theoretical framework | | |
| 9. Methodological orientation and  Theory | What methodological orientation was stated to underpin the study e.g. grounded theory,  discourse analysis, ethnography,  phenomenology, content analysis? | 9 |
| Participant selection | | |
| 10. Sampling | How were participants selected e.g. purposive, convenience, consecutive, snowball? | 5 |
| 11. Method of approach | How were participants approached e.g. face-to- face, telephone, mail, email? | 6 |
| 12. Sample size | How many participants were in the study? | 6,10 |
| 13. Non-  participation | How many people refused to participate or dropped out? Reasons? | 6 |
| Setting | | |
| \| 14. Setting of data collection \| Where was the data collected e.g. home, clinic, workplace? \| 6 \| \| --- \| --- \| --- \| \| 15. Presence of non-participants \| Was anyone else present besides the participants and researchers? \| 8 \| \| 16. Description of sample \| What are the important characteristics of the sample? e.g. demographic data, date \| 10 \| \| Data collection \| \| \| \| 17. Interview guide \| Were questions, prompts, guides provided by the authors? Was it pilot tested? \| 9 \| \| 18. Repeat interviews \| Were repeat interviews carried out? If yes, how many? \| 8 \| \| 19. Audio/visual recording \| Did the research use audio or visual recording to collect the data? \| 7 \| \| 20. Field notes \| Were ﬁeld notes made during and/or after the interview or focus group? \| 7 \| \| 21. Duration \| What was the duration of the interviews or focus group? \| 8 \| \| 22. Data  saturation \| Was data saturation discussed? \| 5,8 \| \| 23. Transcripts returned \| Were transcripts returned to participants for comment and/or correction? \| 7 \| \| Domain 3: Analysis and findings \| \| \| \| Data analysis \| \| \| \| 24. Number of data coders \| How many data coders coded the data? \| 9 \| \| 25. Description of the coding tree \| Did authors provide a description of the coding tree? \| 9 \| \| 26. Derivation of themes \| Were themes identified in advance or derived from the data? \| 9 \| \| 27. Software \| What software, if applicable, was used to manage the data? \| 9 \| \| 28. Participant checking \| Did participants provide feedback on the findings? \| 9 \| \| Reporting \| \| \| \| 29. Quotations presented \| Were participant quotations presented to  illustrate the themes/ findings? Was each  quotation identified? E.g. participant number \| 10-18 \| \| 30. Data and findings  consistent \| Was there consistency between the data presented and the findings? \| 10-18 \| \| 31. Clarity of major themes \| Were major themes clearly presented in the findings? \| 10-18 \| \| 32. Clarity of minor themes \| Is there a description of diverse cases or discussion of minor themes? \| 10-18 \| | | |
